# T cell independent secondary antibody responses to the envelope protein of simian immunodeficiency virus

**DOI:** 10.1186/1742-4690-9-42

**Published:** 2012-05-14

**Authors:** Ghulam Nabi, Vladimir Temchura, Claudius Großmann, Matthias Tenbusch, Klaus Überla

**Affiliations:** 1 Department of Molecular and Medical Virology, Ruhr University Bochum, D-44780, Bochum, Germany; 2 Immune Biology of Retroviral infections, VB, CCR, NCI, NIH, Bethesda, USA

**Keywords:** SIV, HIV, Adenoviral vectors, T-independent antibody response, VLP

## Abstract

**Background:**

During human (HIV) and simian (SIV) immunodeficiency virus infection, loss of CD4+ T cells and progression to AIDS are associated with a decline in antibody titers to the viral Gag protein, while antibodies to the Env protein remain high, suggesting a T cell independent antibody response to Env.

**Results:**

To explore differential regulation of Gag and Env antibody responses, immunocompetent BALB/c and T cell deficient nude mice were immunized with virus like particles (VLP) of simian immunodeficiency virus or adenoviral vectors expressing SIV Gag and Env. High levels of antibodies against Gag and Env could only be induced in immunocompetent mice, but not in the immunodeficient mice. Thus, neither cells expressing Env after adenoviral gene transfer nor VLPs induce a T cell independent primary anti-Env antibody response. However, secondary B cell responses to Env, but not to Gag, were observed in immunodeficient mice after transfer of primed B cells and boosting with VLPs or adenoviral vectors expressing Gag and Env. This T cell independent secondary antibody response to Env was reduced after stimulation with VLPs modified to contain monomeric membrane bound gp130 surface subunit of Env and undetectable after injection of soluble gp130.

**Conclusions:**

Membrane-bound trimeric Env seems to be responsible for the maintenance of high levels of anti-Env antibodies during progression to AIDS. This T cell independent secondary antibody response may prevent T cell-dependent affinity maturation and thus contribute to viral immune escape by favoring persistence of non-protective antibodies.

## Background

Although HIV infection induces a vigorous antibody response to Gag and Env proteins, the induced antibodies do not prevent progression to AIDS. The induction of neutralizing antibodies seems to be too slow and inefficient to keep pace with the rapidly mutating HIV [1,2]. Whether other antibody-mediated antiviral effector mechanisms, such as complement activation and antibody-dependent cytotoxicity, slow down the progression of the disease is unknown [3-5]. The major targets for antibody-mediated inhibition of HIV are the gp120 surface protein (SU), the gp41 transmembrane protein (TM), and possibly the gp160 Env precursor protein. However, the magnitude of the antibody response to Env does not correlate with the slower progression of disease [6-8]. While high levels of Env antibodies are maintained throughout infection, the decline of Gag antibodies with progressing HIV infection is an indicator for a poor prognosis [6-11]. This correlation is unlikely to reflect a direct antiviral activity of Gag antibodies, since Gag proteins are either located inside an infected cell or inside the virus particle with a lipid membrane blocking the accessibility of Gag proteins by antibodies. Macaques chronically infected with simian immunodeficiency virus also maintained high levels of anti-Env antibodies, whereas anti-Gag antibodies declined with progression to AIDS [12,13]. It was, therefore, suggested that the decline of Gag antibodies with progression to AIDS is due to the loss of CD4+ T cell help, while a T cell independent antibody response to Env allows persistence of Env antibodies [6]. A differential regulation of Gag and Env antibody responses was also observed during natural non-progressive infection of African green monkeys. While anti-Gag antibody responses were weak or not observed at all, the anti-Env antibody responses were as robust as observed in HIV infection [14]. Since the limitation of immune activation has been proposed to be a key determinant of non-pathogenic immunodeficiency virus infections [15], the paucity of the Gag-specific antibodies might be due to a limited T helper cell activation. A T cell-independent Env antibody response might then explain the high levels of Env antibodies observed.

The molecular mechanisms mediating the differential requirement of Gag and Env antibodies for T cell help have not been unraveled. Presentation of trimeric Env in a repetitive manner on the surface of virus particles or infected cells might allow cross-linking of Env-specific B cell receptors providing the first signal during B cell activation. However, a T cell independent antiviral antibody response seems to depend on the precise arrangement of the viral surface protein on the viral particle. Infection with vesicular stomatitis virus, which forms viral particles with densely packed G protein spikes, induces T cell independent antibody responses. In contrast, antibody responses to infection with lymphocytic choriomeningitis virus, the virions of which contain less densely packed spikes, are T cell dependent [16]. In addition to the particulate nature of the HIV virion, gp120 was found to have direct B cell stimulatory activity [17]. Thus, gp120 SU might provide both cross-linking of surface BCR (signal 1) and an innate stimulus (signal 2). Theoretically, these two signals alone could be sufficient to trigger differentiation of naïve Env-specific B cells into Ig secreting plasma cells [18].

A T cell independent Env antibody response might also play an important role in viral immune escape: the affinity and avidity of T cell independent Env antibodies might be too low for efficient neutralization. Thus, a better understanding of the differential regulation of Gag and Env-specific B cell responses might provide further insights in the pathogenesis of HIV and guide novel strategies in HIV vaccine development. In the present report, we therefore provide a side by side comparison of Env and Gag-specific primary and secondary antibody responses in immunocompetent and T cell deficient mice.

## Results

### Production and characterization of immunogens

During virus infection, the immune system encounters viral proteins either in the form of infected cells, viral proteins released from infected cells, or viral particles. Since it is not known which of these forms of antigens actually shape the B cell response to immunodeficiency viruses, we wanted to mimic these different forms of viral antigens in a mouse model. Since mice are not susceptible to immunodeficiency virus replication, we reasoned that injection of adenoviral vectors encoding Gag-Pol and Env into mice should result in vector transduced cells producing and releasing the viral proteins in a similar antigenic form as immunodeficiency virus infected cells.

**Figure 1:**
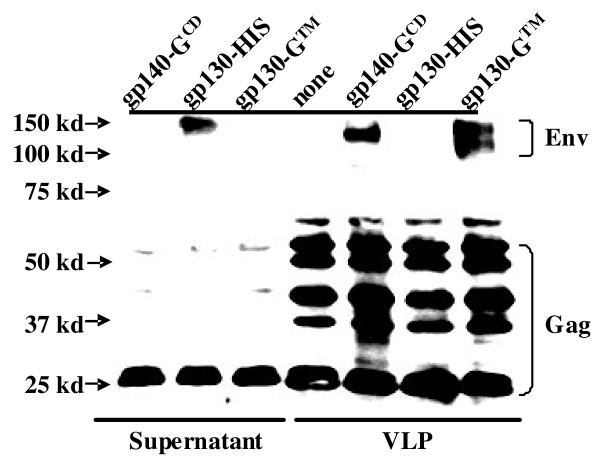
**Western blot analyses.** 293 T cells were co-transfected with Sgp^syn^ and the indicated Env expression plasmids. Supernatants of transfected cells (left) and VLPs partially purified and concentrated by ultracentrifugation (right) were analysed by Western blot for SIV proteins.

To make sure, that the murine immune systems also encounters sufficient amounts of virions, virus-like particles were also prepared *ex vivo* and used as immunogens. In comparison to natural immunodeficiency virus infections which leads to production of over 10^10^ viral particles each day [19,20], the amount of Env and Gag of the VLPs or the amount of Env and Gag produced after adenoviral vector immunization is probably small. To be able to further extend the planned mouse studies into a more relevant animal model for the pathogenesis of AIDS, we used two previously described adenoviral vectors encoding Gag-Pol and Env of SIV [21]. The virus-like particles of SIV were produced by transient co-transfection of 293 T cells with codon-optimized *gag-pol* and *env* expression plasmids. To enhance incorporation of SIV Env into the VLPs, the expression plasmid gp140-G^CD^ was constructed in which the coding region of the intracytoplasmic domain of SIV is replaced by the G protein of vesicular stomatitis virus. Western blot analysis revealed that SIV Env could be detected in VLPs concentrated from the supernatant of gp140-G^CD^-transfected cells by ultracentrifugation through a 20% sucrose cushion, but not in the unconcentrated supernatant of these cells (Figure 1). In contrast, the supernatant of cells, transfected with an expression plasmid encoding the secreted gp130 surface subunit of SIV (gp130-His) contained detectable levels of the Env protein, while the VLP preparation did not (Figure 1).

### Induction of humoral immune responses against Gag and Env

**Figure 2:**
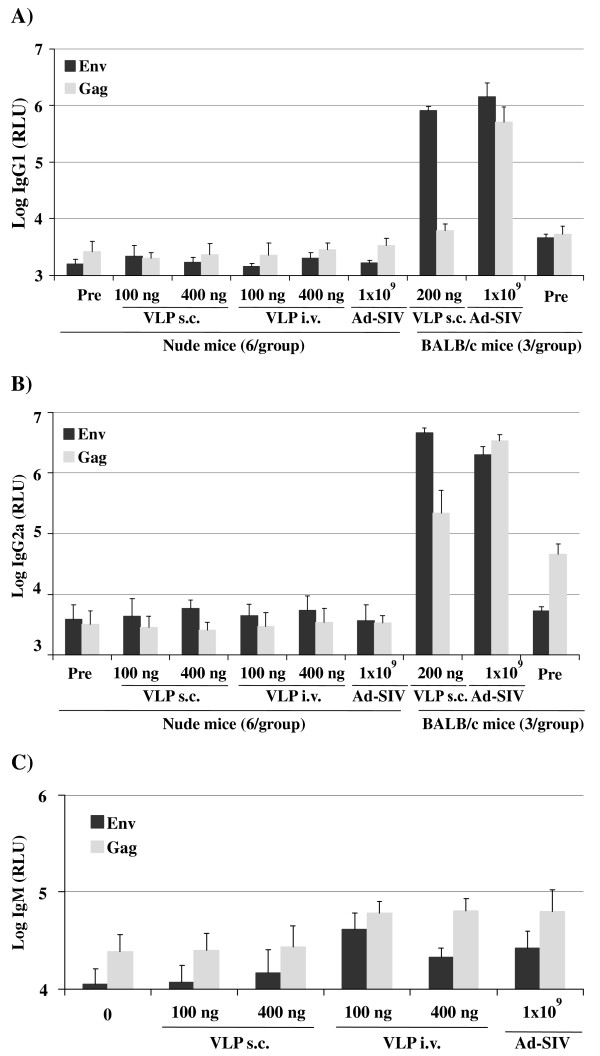
**Antibody response to VLP and adenoviral vector immunization in immunodeficient (A-C) and immunocompetent (A,B) mice.** Mice were immunized with SIV VLPs at the indicated dose of gp140 ectodomain (ng) and route on days 0, 35 and 45. Subcutaneous adenoviral vector immunizations (Ad-SIV) with 1 × 10^9^ particles were performed on day 0 and 35. Serum antibody levels of the indicated isotypes (A-C) to Env and Gag were determined before immunization (0) and on day 49.

To confirm that the VLPs and the adenoviral vectors were immunogenic, immunocompetent mice were immunized subcutaneously with VLPs containing 200 ng of Env or with Ad-SIV, a one-to-one mixture of the two adenoviral vectors encoding Gag-Pol and Env (Figure 2). After two injections of Ad-SIV, mice raised IgG1 and IgG2a antibodies to Gag and Env. Three VLP immunizations induced similar levels of IgG1 and IgG2a antibodies to Env. However, Gag antibody responses were 10- to 100-fold weaker in the VLP immunization group than in the adenoviral vector group, which is consistent with poor accessibility of Gag inside the virus particle.

T cell deficient nude mice were injected in parallel with the same immunogens to explore potential differences in the T-cell independent antibody response to Gag and Env. Immunization of nude mice with VLPs (100 or 400 ng of Env) by either subcutaneous or intravenous injection did not raise Gag and Env specific antibody levels above the background levels seen in pre-immune sera (Figure 2 A,B). The nude mice were immunized with a wider dose range of VLPs to exclude the possibility that passing a narrow threshold level may turn a T cell dependent antibody response into a T cell independent response as observed previously [22]. Subcutaneous immunization with Ad-SIV did not induce Gag- or Env-specific antibodies (Figure 2A,B) either. Since the T cell deficient mice were not able to generate IgG1 and IgG2a antibody responses, we also investigated IgM antibody levels in the immunodeficient mice. Gag and Env-specific IgM antibodies were indeed induced at low levels after intravenous injection of the VLPs and after the adenoviral vector immunization (Figure 2C). Thus, priming of Env- and Gag-specific IgG antibody responses by VLPs and adenoviral vectors greatly depends on T cells.

### T cell independent secondary antibody responses against Gag and Env

**Figure 3:**
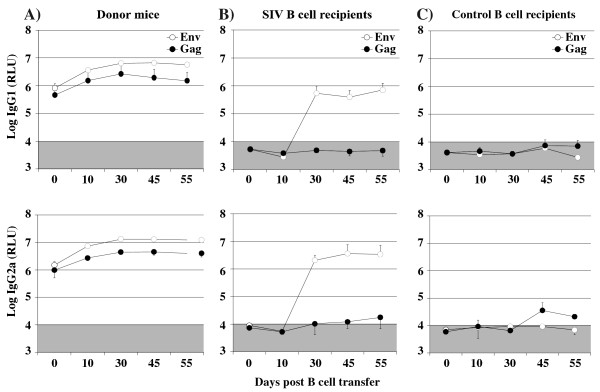
**T cell independent secondary Env antibody responses after adoptive B cell transfer.** BALB/c donor mice were immunized subcutaneously with 1 × 10^9^ particles of Ad-SIV or as a control Ad-GFP. Six weeks later splenic B cells from both groups were transferred to nude mice resulting in SIV B cell recipient and control B cell recipient mice. At 6, 11 and 41 days after B cell transfer, primed B cell recipient, control B cell recipient, and Ad-SIV immunized donor mice were boosted i.v. with VLPs containing 300 ng of the gp140 ectodomain. IgG1 and IgG2a antibody levels to Env and Gag were determined at the indicated time points after B cell transfer. The mean and standard deviation of three animals per group are given. The shaded area indicates background antibody levels.

Persistence of antibodies to Env, but not Gag during progression to AIDS in HIV-infected individuals or SIV-infected macaques suggested a T cell independent Env-specific antibody response, which was clearly not observed during immunization of T cell deficient mice. One major difference between the nude mouse model and natural immunodeficiency virus infections is that, in the latter, priming occurs in immunocompetent hosts. To explore, whether secondary antibody responses to Env are T cell independent, B cell responses were first induced in immunocompetent mice by immunization with adenoviral vectors expressing SIV Gag and Env (Figure 3A). As a control for nonspecific immune activation by the adenoviral vector, a group of mice received an adenoviral vector expressing GFP. Six weeks after adenoviral vector immunization, splenic B cells were isolated and transferred into T cell deficient nude mice. These were then immunized with VLPs 6, 11, and 41 days after the B cell transfer. After the second VLP injection, nude mice that received Gag and Env-primed B cells mounted substantial Env-specific IgG1 and IgG2a antibody levels (Figure 3B). The Env specific antibody response observed in the nude mice was not due to *de novo* priming of B cells since transfer of B cells from mice immunized with the adenoviral GFP vector did not result in Env-specific antibody responses in the recipient nude mice after VLP immunization. Gag-specific secondary antibody responses could not be detected after VLP booster immunizations in the recipient nude mice even after transfer of Gag-primed B cells (Figure 3C).

**Figure 4:**
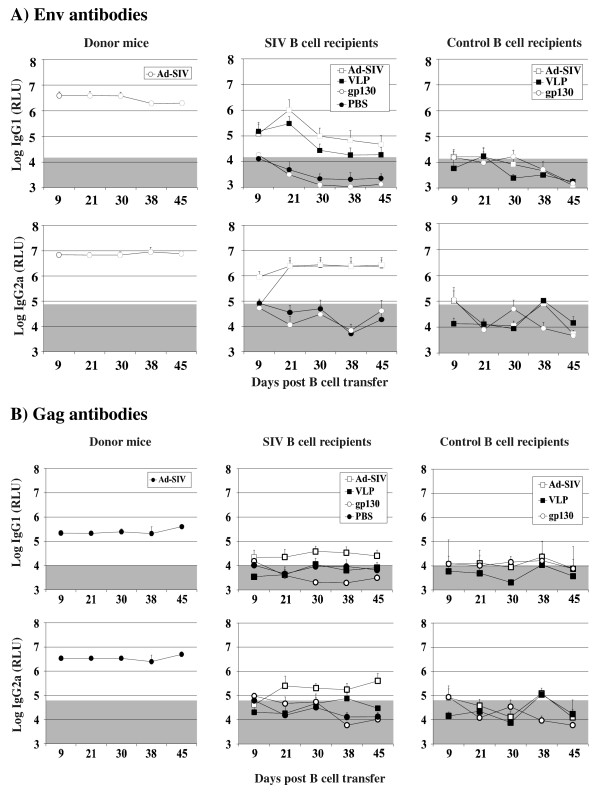
**T cell independent secondary Env antibody responses after adoptive B cell transfer and stimulation with soluble gp130.** BALB/c donor mice were immunized subcutaneously with 1 × 10^9^ particles of Ad-SIV or as a control Ad-GFP. Six weeks later splenic B cells from both groups were transferred to nude mice resulting in SIV B cell recipient and control B cell recipient mice. At 4, 13 and 25 days after B cell transfer, SIV B cell recipient, control B cell recipient and Ad-SIV immunized donor mice were boosted i.v. with VLPs containing 300 ng of the gp140 ectodomain or with 300 ng of secreted gp130 SU. B cell recipient mice were also immunized s.c with Ad-SIV (10^9^ particles, immunization of day 13 ommitted). IgG1 and IgG2a antibody levels to Env (A) and Gag (B) were determined at the indicated time points after B cell transfer. The mean and standard deviation of four animals per group are given. The shaded area indicates background antibody levels.

Since our VLP immunization induced only poor Gag-specific antibody responses even in immunocompetent mice, it remained unclear, whether the absence of Gag-specific secondary antibody responses was due to their T cell dependence or due to poor accessibility of Gag inside the viral particle. To overcome this limitation, nude mice were also immunized with Ad-SIV after adoptive transfer of primed B cells (Figure 4A,B).

Nude mice, that had received Gag and Env-primed B cells developed Gag-specific antibodies only after immunization with the adenoviral vector, but not after VLP injection (Figure 3B). However, the Gag-specific antibody levels were just above background and substantially lower than the Env-specific antibody levels (Figure 4A). This experiment once more confirmed poor induction of Gag-specific secondary antibody responses, while Env-specific secondary immune responses are strongly induced by both the Ad-SIV and VLP immunization indicating that secondary antibody responses to Env are less dependent on T cell help than Gag-specific secondary antibody responses.

### Structural requirements for T cell independent secondary antibody responses to env

The functional Env spikes of human and simian immunodeficiency viruses are formed by cleavage of a trimer of the gp160 Env protein into three SU and three transmembrane TM subunits, which stay non-covalently linked. Expression of SU in the absence of TM leads to secretion of monomeric SU proteins which can be purified from the supernatant of transfected cells (Figure 1). To explore whether the monomeric SU is sufficient to trigger T cell independent secondary Env responses, one group of nude mice, which had received Gag and Env-primed B cells, was also boosted with secreted gp130 SU (Figure 4). Western blot analyses of supernatants of transfected cells revealed the presence of gp130 SU in a form that could not be pelleted through a 20% sucrose cushion (Figure 1). The amount of secreted gp130 SU used for the immunization of the B-cell recipient nude mice was adjusted to the total gp130 content of the VLP preparations.

In contrast to the boost with VLPs or the adenoviral vectors, secreted gp130 SU did not trigger secondary Env antibody responses in T cell deficient mice (Figure 4B). This suggests that the repetitive arrangement of Env on the surface of VLPs or Env-expressing cells is required. Alternatively, the trimeric structure of the envelope spike could trigger B cell receptor cross-linking leading to T cell independent secondary Env antibody responses. We therefore generated VLPs containing a membrane anchored form of *bona fide* monomeric gp130 SU. This was achieved by fusion of the gp130 SU subunit to the transmembrane domain of the G protein of vesicular stomatitis virus. Western blot analyses confirmed that this membrane anchored form of gp130 was no longer secreted at detectable levels into the supernatant of transfected cells (Figure 1). In contrast to the secreted gp130, it could be pelleted by ultracentrifugtion through a 20% sucrose cushion (Figure 1) suggesting efficient incorporation into VLPs.

**Figure 5:**
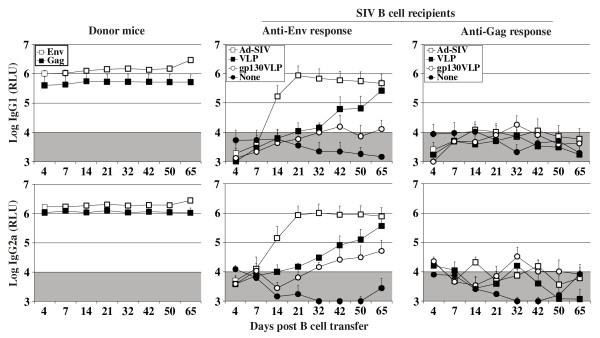
**T cell independent secondary Env antibody responses after adoptive B cell transfer and stimulation with VLPs containing monomeric gp130 SU.** BALB/c donor mice were immunized subcutaneously with 1 × 10^9^ particles of Ad-SIV. Six weeks later, splenic B cells were transferred into nude mice resulting in SIV B cell recipient mice. At 4, 35 and 41 days after B cell transfer, SIV B cell recipient and Ad-SIV immunized donor mice were boosted i.v. with VLPs containing 300 ng gp140 ectodomain. B cell recipient mice were also immunized s.c with Ad-SIV (10^9^ particles, immunization of day 35 omitted) or i.v. with VLPs containing 300 ng of membrane-anchored monomeric gp130 (gp130-VLPs). IgG1 and IgG2a antibody levels to Env and Gag were determined at the indicated time points after B cell transfer. The mean and standard deviation of four animals per group are given. The shaded area indicates background antibody levels.

Boosting nude mice that had received Gag- and Env-primed B cells with gp130-VLPs triggered secondary Env-specific IgG1 and IgG2a antibody responses that were approximately 2 to 20-fold lower than those obtained after a boost with VLPs containing the same amount of the wild type ectodomain of Env (Figure 5). This indicates that the level of T cell independent Env-specific secondary antibody response depends on the repetitive arrangement of Env on the surface of infected cells or on VLPs. In comparison to membrane-bound monomeric form of gp130 SU, the trimeric nature or a particular conformation of the native Env spike seems to further increase T cell independent induction of the secondary Env antibody responses.

## Discussion

HIV-1 infection leads to an early and vigorous humoral immune response. Antibodies to gp120 SU and gp4l TM become detectable first, but are followed shortly by antibodies to Gag [23]. Sequential serum samples from HIV-infected patients show a decline of antibodies to Gag and Tat coinciding with more rapid progression to AIDS [24-26]. This decline of the anti-Gag antibody response during disease progression has been proposed to reflect the loss of T cell help [6,27]. The maintenance of high levels of anti-Env antibodies despite declining anti-Gag antibody levels indicates a differential requirement for CD4^+^ T cell help. Similar observations were made in rhesus monkeys experimentally infected with SIV [12,13]. Since several spikes of the trimeric complexes are presented on the surface of virions and thus provide repetitive antigenic determinants, Env indeed possesses some characteristics of a T-cell independent antigen [28].

Therefore, we hypothesized that nude mice should be able to elicit anti-Env antibody responses in a T cell independent manner. It was previously reported that immunization of CD4KO mice with HIV-1 VLPs produced by a baculovirus expression system in insect cells induced Env-specific antibodies in mice independently from T-helper cells [29]. However, co-purification of baculoviruses with the VLPs is suspected to provide substantial adjuvant effects [30], which limits the conclusions that can be drawn with respect to T cell-independent antibody responses induced by the lentiviral VLPs itself. In our hands the immunization of nude mice with Ad-SIV or SIV-VLPs produced in a human cell line did not raise Env- or Gag-specific IgG immune responses, although both immunization regimens proved to be immunogenic in immunocompetent BALB/c mice (Figure 2A,B). This indicates that the primary antibody response to SIV Env is T cell dependent in mice. However, we cannot exclude that the lack of T cell independent primary Env antibody responses in mice is due to species-specific differences in the innate interaction of Env with B cells, which warrants further investigations.

The longitudinal analyses of immune responses during HIV and SIV infection suggest that the anti-Env antibody response is maintained in a T cell independent manner. This hypothesis could be confirmed by our mouse experiments. After adoptive transfer of B cells from SIV immunized immunocompetent mice into nude mice, exposure of the recipient mice to VLPs or Ad-SIV resulted in an Env-specific IgG antibody response, whereas anti-Gag antibody responses were not elicited (Figure 3, 4, 5). Since we observed the Env antibody responses only in T cell deficient nude mice receiving primed B cells, the detected antibody response must be T cell independent. Neither the autologous B cells of nude mice nor B cells transferred from Ad-GFP immunized mice can raise an Env antibody response indicating that the primary antibody response is T cell dependent.

In our transfer experiments, B cells were obtained from the spleens of the primed BALB/c mice six weeks after single Ad-SIV immunization. Since the transferred B cells did not produce Env- or Gag-specific antibodies in recipient nude mice without antigen re-stimulation (Figure 3), they should be predominantly composed of antigen-specific memory B cells [31-33]. Memory B cells circulate as a backup system and provide polyclonal maintenance. Upon re-exposure to the same antigen, they differentiate into new plasma cells leading to a rapid recall response [32,33].

In all the B cell transfer experiments in nude mice, anti-Gag humoral immune responses were not observed after antigen re-stimulation. However, we could demonstrate T cell independent differentiation of Env-specific memory B cells into IgG producing plasma cells probably by the direct engagement of the BCR. Although antibody production is believed to be dependent on delivery of a second signal, in the absence of T cell help, the second signal could be potentially provided by other signaling pathways including those mediated *via* pattern recognition receptors [34]. The HIV envelope was shown to trigger human B cells through a CD40-independent mechanism involving innate BAFF and mannose C-type lectin receptors [17]. Thus, simultaneous activation of antibody-producing B cells *via* this innate mechanism and the extensive cross-linking of BCRs by such multivalent ligands as the Env spikes might itself be sufficient to induce proliferation of B cells, as previously discussed [35].

Our mouse model also allowed us to explore the structural requirements for the T-cell independent secondary antibody responses to Env. Each of the Env spikes of human and simian immunodeficiency viruses consists of three SU and TM subunits, which are non-covalently linked and embedded in the cytoplasmic membrane of infected cells and circulating virions. At the same time, SU is constantly shedded in a monomeric form. Therefore, we determined whether the secreted SU can also trigger T-cell-independent secondary antibody responses. The secreted monomeric gp130 SU clearly failed to trigger secondary antibody responses, whereas the same amounts of gp130 incorporated into VLPs within the wild type multimeric Env spikes induced good secondary IgG antibody responses to Env (Figure 4).

To answer the question whether the T-cell-independent secondary antibody is elicited by the trimeric nature of Env or is due to the repetitive nature of the Env-spikes, we immunized B cell recipient nude mice with VLPs containing membrane-bound monomeric form of Env. The secondary antibody response to Env after stimulation with VLPs containing monomeric membrane-bound SU was detectable, but it was lower in magnitude than the antibody response after stimulation with VLPs containing trimeric forms of Env (Figure 5). The repetitive nature of the membrane-anchored monomeric SU probably helps to cross-link multiple B cell receptors resulting in the stimulation of anti-Env antibody response. However, the higher magnitude of antibody response after immunization with VLP containing trimeric Env suggests that the Env trimers are even more efficient in BCR cross-linking and/or providing the second signal.

Although there are numerous reports indicating that the generation of plasma cells from virus-specific memory B cell is a strictly T cell-dependent immune reaction [31,32,36], there is another publication demonstrating that T-cell-independent humoral immune responses to enveloped viruses can occur. Using adoptive transfer of memory B cells from immunocompetent mice immunized with human cytomegalovirus into RAG-1^−/−^ animals, Hebeis and colleagues observed T cell independent activation of memory B cells specific for viral surface proteins after challenge of the B-cell recipient mice with the same virus [37]. As suggested by the authors, this T cell independent antiviral memory B cell activation could be particularly beneficial during reactivation of viruses in immunosuppressed patients [37,38]. However, the proposed T cell independent activation mechanism could be a serious disadvantage for the host during infection with viruses with high mutation rates. After infection of a host, HIV continues to accumulate mutations in B cell epitopes targeted by neutralizing antibodies. Sera from HIV-infected patients were unable to neutralize HIV isolated from the same donor at the time of serum sampling, but could readily neutralize isolates obtained at previous sampling time points [39,40]. Since there seems to be a competition between newly generated plasmablasts and established resident long-lived plasma cells for habitation of a limited number of survival niches [33], the B cell responses to past variants of Env might therefore interfere with timely generation of neutralizing antibodies targeting the escape mutants. T cell independent Env antibody production might therefore contribute to the immune escape of immunodeficiency viruses from antibody mediated immune mechanisms.

## Conclusions

Our findings are consistent with the hypothesis that impairment of T cell help during progression to AIDS leads to decline in effective plasma cells generation from Gag-specific memory B cells established during the initial phase of HIV infection. Therefore, the anti-p24 antibody response decays with time in the advanced stages of the disease [6,41]. Our findings further suggest that trimeric membrane-associated forms of Env are able to continuously stimulate generation of Env-specific plasma cells even upon total loss of cognate help.

## Methods

### Plasmids and recombinant adenoviruses

The codon-optimized SIV-gag-pol expression plasmid Sgp^syn^ has been previously described [42]. Codon-optimized fragments (obtained from Geneart, Regensburg, Germany) encoding amino acids 23 to 682 (gp140 ectodomain and TM domain) or 23 to 521 (gp130 SU) of SIVmac239 *env* (according to GenBank entry M33262.1) were cloned into pcDNA3.1+ (Invitrogen) downstream of the coding region for the leader peptide of tissue plasminogen activator (amino acid 1 to 23 of GenBank: AAA61213.1). A codon-optimized fragment encoding the cytoplasmic domain of the G-protein of VSV (amino acid 97 to 122 of GenBank: CAA24524.1 and derived from plasmid pCD-Gsynmut [43]) was fused in frame to the gp140 ectodomain construct to generate plasmid gp140-G^CD^. A codon-optimized fragment encoding the transmembrane and cytoplasmic domains of the G-protein of VSV (amino acid 52 to 122 of GenBank: CAA24524.1) was fused in frame to the gp130 construct to generate plasmid gp130-G^TM^. Fusing the coding region for the 6xHis-tag to the gp130 SU construct resulted in plasmid gp130-His. A codon-optimized fragment spanning SIV p27CA (Gag amino acid 135 to 363 of Genbank entry M33262.1) was also fused in frame with a coding region for a 6xHis-tag and cloned into pET15b vector (Novagen) to generate the SIVp27pET15b plasmid.

The construction and production of the adenoviral vectors expressing codon-optimized SIV *gag-pol* (Ad-Sgp^syn^), codon-optimized SIV *env* (Ad-Senv-co), and GFP (Ad-GFP) have been described previously [44]. Ad-SIV refers to a one-to-one mixture of Ad-Sgp^syn^ and Ad-Senv-co.

### VLP production and characterisation

HEK 293 T cells were cultured in DMEM (Invitrogen, Karlsruhe, Germany) supplemented with penicillin, streptomycin and 10% FCS. Virus like particles containing the wild type ectodomain (VLP) or the membrane-bound surface subunit of Env (gp130VLP) were produced in 293 T cells by transient cotransfection of 293 T cells with Sgp^syn^ and gp140-G^CD^ or gp130-G^TM^ respectively. Eight hours after transfection, the FCS-containing DMEM was replaced by serum-free AIM-V medium (Invitrogen). The conditioned medium was harvested 24 h later and the VLPs were concentrated by ultracentrifugation through 20% sucrose in Optima L-70 K, Beckman ultracentrifuge at 25000 rpm for 2 h using a SW28 rotor as described previously [45]. The pellet was resuspended in approximately 200 μl PBS per 30 ml supernatant of transfected cells and stored at −80°C for subsequent experiments. Secreted SIV gp130 was purified from the supernatant of gp130-His-transfected 293 T cells using the ProPur Midi MC kit (Nunc, Germany).

The Env and Gag content of the immunogens was determined by coating ELISA plates with the SIV gp130 SU preparation or with VLP preparations lysed in 0.2% Triton X-100. Env was then detected by the KK45 monoclonal antibody directed against SIVmac251 gp120 (NIH AIDS research and reference reagent program) and Gag proteins by an HIV-1 p24 specific monoclonal antibody, purified from the hybridoma 183-H12-5 C (NIH AIDS research and reference reagent program). Binding of these antibodies was detected by a peroxidase-labelled secondary antibody. Defined amounts of recombinant SIV gp130 SU (EVA670, NIBSC) and SIV p27CA (EVA643) were coated and analysed in parallel to obtain standard curves. The endotoxin levels of the immunogens were determined by the Limulus assay kit (QCL-1000® Chromogenic LAL Endpoint Assay, Cambrex) and it was made sure that the final endotoxin levels remained below 0.1 units per injection dose.

### Animal experiments

Immunodeficient female nude mice (BCAnN.Cg-*Fox1nu*/Crl) and immunocompetent female BALB/c mice, 5 to 7 weeks old, were purchased from Charles River Laboratories and housed in the central animal facility of the Medical Faculty of the Ruhr-University. Animal care and use were in compliance with the German Animal Protection Law. Approval for the animal experiments was obtained from the Landesamt für Natur, Umwelt und Verbraucherschutz Nordrhein-Westfalen (reference number 50.8735.1. Nr. 111/2). For subcutaneous injections, 50 μl of each immunogen were injected into both hind foot pads, a total volume of 300 μl was injected for intravenous administration of VLPs in the lateral tail vein after warming the mice with infrared heating lamp for 40 to 60 seconds. Blood samples were obtained by puncture of the retro-orbital plexus under isoflurane inhalation anaesthesia of the mice.

For the B-cell transfer experiments, BALB/c mice were immunized with Ad-SIV or Ad-GFP (1x10^9^ particles, s.c). On week 6 after the immunization, the B cells were isolated from spleens by the B cell isolation kit (Miltenyi Biotec GmbH**).** The purity of isolated cells reached 96–98% as confirmed by flow cytometry. B cells were pooled and the approximate number of B cells purified per mouse was injected intravenously into each recipient nude mouse. Each recipient group had four mice.

### Measurement of serum Ab level (ELISA)

The Gag antigen (p27CA) for the ELISA was produced in BL21(DE3) cells transformed by SIVp27pET15b as suggested by the manufacturer (Stratagene). The protein was purified from the periplasm by its His-tag using the ProPur Midi MC kit (Nunc, Denmark) according to the manufacturer’s instructions. The protein preparations were 90–95% pure as judged by Coomassie staining. To determine serum antibody levels to Env or Gag flat-bottom 96-well plates (Nunc) were coated overnight with 200 ng p27CA or secreted SIVgp130 produced in 293 T cells prior to blocking with 5% fat-free milk. After washing, the wells were incubated with dilutions of the sera from immunized mice and bound IgG1 and IgG2a antibodies were detected using HRP-conjugated secondary antibodies obtained from Sigma Aldrich. After adding the enhanced chemiluminescent substrate the relative light units emitted were determined in a microplate luminometer. Serum dilutions within the dynamic range of the ELISA were used.

## Competing interests

The authors declare that they have no competing interests.

## Authors’ contributions

GN constructed vectors encoding SIV antigens, participated in the study design, carried out the experiments and drafted the manuscript. VT assisted in isolating the B cells from spleens and injecting them into recipient nude mice. CG helped in cloning the G^CD^ vector. SK provided the adenoviral vectors expressing GFP, SIV env and Gag. MT performed flow cytometric analyses to ascertain the purity of isolated B cells and helped in mice experiments. KÜ conceived the study, participated in its design, and wrote the manuscript. All authors read and approved the final manuscript.
